# A second polymorph of *catena*-poly[[(1,10-phenanthroline-κ^2^ *N*,*N*′)copper(II)]-di-μ-thio­cyanato-κ^2^ *N*:*S*;κ^2^ *S*:*N*]

**DOI:** 10.1107/S1600536811001759

**Published:** 2011-03-05

**Authors:** Shi-Shen Zhang, Li-Jiang Chen, Yi-Feng Han

**Affiliations:** a Department of Applied Chemistry, Zhejiang Sci-Tech University, Hang Zhou 310018, People’s Republic of China, and Engineering Research Center for Eco-Dyeing & Finishing of Textiles, Ministry of Education, Zhejiang Sci-Tech University, Hangzhou 310018, People’s Republic of China

## Abstract

In the title coordination polymer, [Cu(NCS)_2_(C_12_H_8_N_2_)]_*n*_, the Cu^II^ atom is situated on a twofold rotation axis and is coordinated by two N atoms from the bidentate 1,10-phenanthroline ligand and four thio­cyanate groups to confer a CuN_4_S_2_ octa­hedral geometry and resulting in a layer structure extending parallel to (100).

## Related literature

For the first polymorph of this composition, see: Breneman & Parker (1993 ▶). For related structures, see: Kulkarni *et al.* (2002 ▶); Morpurgo *et al.* (1984 ▶).
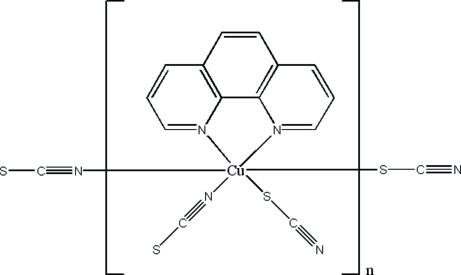


## Experimental

### 

#### Crystal data

[Cu(NCS)_2_(C_12_H_8_N_2_)]
*M* *_r_* = 359.90
Monoclinic, 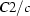

*a* = 14.0353 (13) Å
*b* = 10.3081 (9) Å
*c* = 10.2670 (9) Å
β = 111.034 (2)°
*V* = 1386.4 (2) Å^3^
*Z* = 4
Mo *K*α radiation
μ = 1.87 mm^−1^
*T* = 294 K
0.25 × 0.22 × 0.15 mm

#### Data collection

Bruker SMART diffractometer
Absorption correction: multi-scan (*SADABS*; Sheldrick, 1996 ▶) *T*_min_ = 0.633, *T*_max_ = 0.755
3938 measured reflections
1362 independent reflections
1254 reflections with *I* > 2σ(*I*)
*R*_int_ = 0.015

#### Refinement

*R*[*F* ^2^ > 2σ(*F* ^2^)] = 0.027
*wR*(*F* ^2^) = 0.076
*S* = 1.08
1362 reflections
97 parameters
H-atom parameters constrained
Δρ_max_ = 0.33 e Å^−3^
Δρ_min_ = −0.31 e Å^−3^

### 

Data collection: *SMART* (Bruker, 1998 ▶); cell refinement: *SAINT* (Bruker, 1998 ▶); data reduction: *SAINT*; program(s) used to solve structure: *SHELXS97* (Sheldrick, 2008 ▶); program(s) used to refine structure: *SHELXL97* (Sheldrick, 2008 ▶); molecular graphics: *SHELXTL* (Sheldrick, 2008 ▶); software used to prepare material for publication: *SHELXTL*.

## Supplementary Material

Crystal structure: contains datablocks I, global. DOI: 10.1107/S1600536811001759/ng5084sup1.cif

Structure factors: contains datablocks I. DOI: 10.1107/S1600536811001759/ng5084Isup2.hkl

Additional supplementary materials:  crystallographic information; 3D view; checkCIF report

## Figures and Tables

**Fig. 1.**
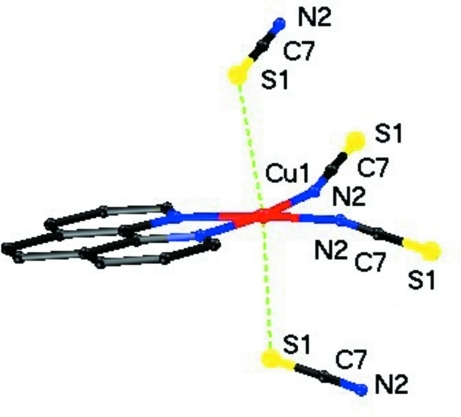
The coordination environment of the title compound

**Fig. 2.**
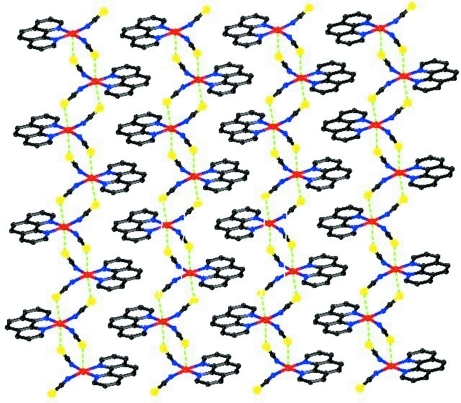
The crystal packing of the title compound

## supplementary crystallographic information

### Comment

Phenanthroline and its derivatives have been achieving rapidly increasing attention not only for their potential application as functional materials, but aslo from their intriguing variety of architectures and topologies. 1, 10-Phenanthroline, as one kind of those ligand, has usually been used to construct a great variety of structurally interesting entities, such as monomers(Breneman *et al.* 1993), ploymers(Kulkarni *et al.* 2002; Morpurgo *et al.* 1984).

The structure of the title compound (I) is illustrated in Fig. 1. the Cu^II^ atom is coordinated by two N atoms from1, 10-Phenanthroline ligand, as well as by the two N atoms and two S atoms from four thiocyanate groups to confer a distorted octahedral coordination at the metal centre. Two S atoms occupy the axial position, showing weak interaction of Cu1—S1 bond [2.952 (3)], which give rise to one-dimensional chain along (100), the crystal packing is stabilized by the intermolecular π-π stacking interaction(Fig. 2).

In contrast to the first polymorph of this composition in which the distance of  Cu—S bonds are longer [3.163 (2) Å], and the S—Cu—S' angles are nearly linear [170.86 (6)°].  The S—Cu—N angles in reported complex vary from 73.8 (1) to 99.1 (1)°, which make the octahedral geometry of this compound more disordered than the title compoud.

### Experimental

The mixture of CuSCN (0.0244 g, 0.2 mmol), 1, 10-Phenanthroline (0.0132 g, 0.1 mmol), were placed and sealed in a 10 ml Teflon-lined stainless steel reactor and heated to 160 °C for 72 h, then cooled down to room temperature at a rate of 5 °C/ 60 min. Single crystals suitable for X-ray diffraction were obtained in the form of black bars in *ca* 35% yield.

The web of checkcif show one Alert level B(Hirshfeld Test Diff S1 – C7..8.52 su), we think this is the result of the sightly distorted S atom of the thiocyanate group for his weak interaction to the Cu atom.

### Refinement

H atoms were positioned geometrically and refined using a riding model, with C—H = 0.93?Å(aromatic) and Uĩso(H) = 1.2Ueq(C)

### Crystal data

**Table:**

[Cu(NCS)_2_(C_12_H_8_N_2_)]	*F*(000) = 724
*M**_r_* = 359.90	*D*_x_ = 1.724 Mg m^−^^3^
Monoclinic, *C*2/*c*	Mo *K*α radiation, λ = 0.71073 Å
Hall symbol: -c 2yc	Cell parameters from 1647 reflections
*a* = 14.0353 (13) Å	θ = 2.5–27.8°
*b* = 10.3081 (9) Å	µ = 1.87 mm^−^^1^
*c* = 10.2670 (9) Å	*T* = 294 K
β = 111.034 (2)°	Block, black
*V* = 1386.4 (2) Å^3^	0.25 × 0.22 × 0.15 mm
*Z* = 4	

### Data collection

**Table:**

Bruker SMART diffractometer	1362 independent reflections
Radiation source: fine-focus sealed tube	1254 reflections with *I* > 2σ(*I*)
graphite	*R*_int_ = 0.015
φ and ω scans	θ_max_ = 26.0°, θ_min_ = 2.5°
Absorption correction: multi-scan (*SADABS*; Sheldrick, 1996)	*h* = −17→17
*T*_min_ = 0.633, *T*_max_ = 0.755	*k* = −12→5
3938 measured reflections	*l* = −12→12

### Refinement

**Table:**

Refinement on *F*^2^	Secondary atom site location: difference Fourier map
Least-squares matrix: full	Hydrogen site location: inferred from neighbouring sites
*R*[*F*^2^ > 2σ(*F*^2^)] = 0.027	H-atom parameters constrained
*wR*(*F*^2^) = 0.076	*w* = 1/[σ^2^(*F*_o_^2^) + (0.0475*P*)^2^ + 0.5818*P*] where *P* = (*F*_o_^2^ + 2*F*_c_^2^)/3
*S* = 1.08	(Δ/σ)_max_ = 0.001
1362 reflections	Δρ_max_ = 0.33 e Å^−^^3^
97 parameters	Δρ_min_ = −0.31 e Å^−^^3^
0 restraints	Extinction correction: *SHELXL97* (Sheldrick, 2008), Fc^*^=kFc[1+0.001xFc^2^λ^3^/sin(2θ)]^-1/4^
Primary atom site location: structure-invariant direct methods	Extinction coefficient: 0.0008 (3)

### Special details

**Table:**

Geometry. All e.s.d.'s (except the e.s.d. in the dihedral angle between two l.s. planes) are estimated using the full covariance matrix. The cell e.s.d.'s are taken into account individually in the estimation of e.s.d.'s in distances, angles and torsion angles; correlations between e.s.d.'s in cell parameters are only used when they are defined by crystal symmetry. An approximate (isotropic) treatment of cell e.s.d.'s is used for estimating e.s.d.'s involving l.s. planes.
Refinement. Refinement of *F*^2^ against ALL reflections. The weighted *R*-factor *wR* and goodness of fit *S* are based on *F*^2^, conventional *R*-factors *R* are based on *F*, with *F* set to zero for negative *F*^2^. The threshold expression of *F*^2^ > σ(*F*^2^) is used only for calculating *R*-factors(gt) *etc*. and is not relevant to the choice of reflections for refinement. *R*-factors based on *F*^2^ are statistically about twice as large as those based on *F*, and *R*- factors based on ALL data will be even larger.

### Fractional atomic coordinates and isotropic or equivalent isotropic displacement parameters (Å^2^)

**Table:**

	*x*	*y*	*z*	*U*_iso_*/*U*_eq_	
Cu1	0.5000	0.62211 (3)	0.2500	0.03359 (15)	
N2	0.43016 (13)	0.49319 (16)	0.32227 (17)	0.0407 (4)	
N1	0.56960 (11)	0.77181 (16)	0.19112 (15)	0.0330 (3)	
C7	0.39033 (14)	0.43783 (18)	0.38718 (19)	0.0324 (4)	
C6	0.53802 (14)	0.88917 (17)	0.21873 (19)	0.0326 (4)	
C1	0.64028 (15)	0.7684 (2)	0.1322 (2)	0.0426 (5)	
H1	0.6632	0.6884	0.1136	0.051*	
C4	0.57499 (16)	1.0064 (2)	0.1877 (2)	0.0415 (5)	
C3	0.64909 (17)	0.9994 (2)	0.1252 (2)	0.0488 (6)	
H3	0.6761	1.0749	0.1027	0.059*	
C2	0.68083 (19)	0.8814 (2)	0.0979 (3)	0.0507 (6)	
H2	0.7296	0.8759	0.0562	0.061*	
S1	0.33300 (4)	0.36018 (5)	0.47588 (6)	0.04095 (18)	
C5	0.5358 (2)	1.12518 (19)	0.2202 (3)	0.0548 (6)	
H5	0.5599	1.2038	0.1999	0.066*	

### Atomic displacement parameters (Å^2^)

**Table:**

	*U*^11^	*U*^22^	*U*^33^	*U*^12^	*U*^13^	*U*^23^
Cu1	0.0418 (2)	0.0268 (2)	0.0433 (2)	0.000	0.02887 (17)	0.000
N2	0.0479 (10)	0.0375 (9)	0.0432 (9)	−0.0075 (8)	0.0242 (8)	0.0008 (7)
N1	0.0348 (8)	0.0338 (8)	0.0351 (8)	−0.0009 (6)	0.0182 (6)	0.0016 (6)
C7	0.0353 (9)	0.0279 (9)	0.0358 (9)	−0.0008 (8)	0.0148 (8)	−0.0027 (7)
C6	0.0359 (10)	0.0312 (9)	0.0302 (9)	−0.0021 (7)	0.0111 (8)	0.0015 (7)
C1	0.0424 (10)	0.0461 (12)	0.0487 (11)	−0.0017 (9)	0.0280 (9)	0.0019 (9)
C4	0.0451 (11)	0.0366 (11)	0.0391 (10)	−0.0057 (9)	0.0107 (9)	0.0052 (8)
C3	0.0515 (12)	0.0467 (13)	0.0501 (12)	−0.0150 (10)	0.0206 (10)	0.0097 (10)
C2	0.0481 (12)	0.0637 (16)	0.0498 (13)	−0.0116 (10)	0.0293 (11)	0.0039 (10)
S1	0.0430 (3)	0.0442 (3)	0.0433 (3)	−0.0070 (2)	0.0249 (2)	0.0026 (2)
C5	0.0705 (17)	0.0301 (11)	0.0589 (15)	−0.0062 (9)	0.0174 (12)	0.0025 (9)

### Geometric parameters (Å, °)

**Table:**

Cu1—N2	1.9492 (16)	C1—C2	1.397 (3)
Cu1—N2^i^	1.9492 (16)	C1—H1	0.9300
Cu1—N1	2.0310 (15)	C4—C3	1.406 (3)
Cu1—N1^i^	2.0310 (15)	C4—C5	1.430 (3)
N2—C7	1.162 (3)	C3—C2	1.359 (3)
N1—C1	1.335 (2)	C3—H3	0.9300
N1—C6	1.353 (2)	C2—H2	0.9300
C7—S1	1.6259 (19)	C5—C5^i^	1.351 (6)
C6—C4	1.397 (3)	C5—H5	0.9300
C6—C6^i^	1.430 (4)		
			
N2—Cu1—N2^i^	94.04 (10)	N1—C1—H1	119.0
N2—Cu1—N1	173.03 (6)	C2—C1—H1	119.0
N2^i^—Cu1—N1	92.49 (7)	C6—C4—C3	117.1 (2)
N2—Cu1—N1^i^	92.49 (7)	C6—C4—C5	118.8 (2)
N2^i^—Cu1—N1^i^	173.03 (6)	C3—C4—C5	124.1 (2)
N1—Cu1—N1^i^	81.10 (9)	C2—C3—C4	119.4 (2)
C7—N2—Cu1	164.69 (16)	C2—C3—H3	120.3
C1—N1—C6	118.10 (17)	C4—C3—H3	120.3
C1—N1—Cu1	129.05 (15)	C3—C2—C1	120.1 (2)
C6—N1—Cu1	112.85 (12)	C3—C2—H2	120.0
N2—C7—S1	179.12 (18)	C1—C2—H2	120.0
N1—C6—C4	123.34 (18)	C5^i^—C5—C4	121.13 (13)
N1—C6—C6^i^	116.59 (10)	C5^i^—C5—H5	119.4
C4—C6—C6^i^	120.07 (12)	C4—C5—H5	119.4
N1—C1—C2	121.9 (2)		

Symmetry codes: (i) −*x*+1, *y*, −*z*+1/2.
